# Adjusting intervention strategies for mental health of COVID-19 patients: A network analysis based on a survey in Omicron-infected patients

**DOI:** 10.3389/fpubh.2022.1038296

**Published:** 2022-11-17

**Authors:** Kuiliang Li, Keyong Luo, Xiaoqing Zhan, Chang Liu, Ling Li, Xi Luo, Lei Ren, Lingzhi Wang, Zhengzhi Feng

**Affiliations:** ^1^School of Psychology, Army Medical University, Chongqing, China; ^2^Department of Psychiatry, The 980th Hospital of PLA Joint Logistics Support Force, Shijiazhuang, China; ^3^Department of Medical English, College of Basic Medicine, Army Medical University, Chongqing, China; ^4^BrainPark, Turner Institute for Brain and Mental Health and School of Psychological Sciences, Monash University, Clayton, VIC, Australia; ^5^College of General Education, Chong Qing Water Resources and Electric Engineering College, Chongqing, China; ^6^Department of Psychology, Fourth Military Medical University, Xi'an, China; ^7^Sichuan Shun Dao Law Firm, Chengdu, China

**Keywords:** network analysis, COVID-19, depression, anxiety, perceived stress, patients infected with Omicron

## Abstract

**Background:**

The COVID-19 pandemic had a major impact on people's mental health. As the SAS-Cov-2 evolves to become less virulent, the number of asymptomatic patients increases. It remains unclear if the mild symptoms are associated with mild perceived stress and mental illness, and the interventions to improve the mental health of the patients are rarely reported.

**Methods:**

This cross-sectional study investigated the level of depression, anxiety and perceived stress of 1,305 COVID-19 patients who received treatment in the Fangcang shelter hospitals in Shanghai, China. Network analysis was used to explore the relationship among depression, anxiety and perceived stress.

**Results:**

The prevalence of depression, anxiety and perceived stress in the patients with Omicron infection were 9.03, 4.60, and 17.03%, respectively, lower than the prevalence reported during the initial outbreak of COVID-19. “Restlessness (A5),” “Uncontrollable worry (A2),” “Trouble relaxing (A4)” and “Fatigue (D4)” had the highest expected influence values. “Irritability (A6)” and “Uncontrollable (S1)” were bridge symptoms in the network. Comparative analysis of the network identified differences in the network structures between symptomatic and asymptomatic patients.

**Conclusion:**

This study investigated the prevalence of depression, anxiety and perceived stress and the correlation among them in Omicron-infected patients in Fangcang shelter hospital, in Shanghai, China. The core symptoms identified in the study provide insight into targeted clinical prevention and intervention of mental health in non-severe Omicron-infected patients.

## Introduction

Severe Acute Respiratory Syndrome Coronavirus 2 (SARS-CoV-2) has infected more than 500 million people worldwide in over 200 countries since it was detected in December 2019 in Wuhan, China, causing more than 6 million deaths (1). At present, a variety of mutant strains of this highly infectious virus have developed, and one of the most prevalent strains is Omicron variant. Since Omicron variant (B.1.1.529) was detected and reported in South Africa in November 2021, it has gradually become the dominant strain sweeping the world (2). Recently, a epidemic of Omicron infection broke out in Shanghai, China, infecting about 600,000 people in just a few months (3).

During the COVID-19 pandemic, its associated mental health problems have attracted widespread attention, and the most frequently reported are depression and anxiety (4). In the first year of the outbreak, their prevalence was increased by about 25%, respectively (5).

A previous cross-sectional survey in 2020 in Iran showed that the depression and anxiety levels of COVID-19 patients were as high as 54.29 and 97.29% respectively, and 46.61% reported severe perceived stress (6). The study in Wuhan, China also showed that 44.9 and 24.9% of patients with moderate COVID-19 infection had clinically significant depression and anxiety symptoms respectively (7). Moreover, the depression and anxiety levels of COVID-19 inpatients were higher than those of outpatients. Patients with smell and taste loss had higher anxiety and depression scores both 1 week and 1 month after diagnosis (8).

With the evolution of the virus strain, the symptoms caused by infection alleviated gradually. The longitudinal data analysis of seven European countries showed that, with the development of the epidemic, the prevalence of depressive symptoms decreased by 2.8% and that of anxiety symptoms decreased by 3.6% during the sixth epidemic Wave in April 2021 compared with those in the fourth epidemic wave in November 2020 (9). The reported case fatality rate of Alpha strain broke out in September 2020 in UK remained 1.1% from February to August in 2021, while that of the Delta strain broke out in October 2020 was 0.3%. The risk of serious consequences in patients infected with Omicron variant was much lower than that of Delta variant (10). The patients infected with Omicron mainly experienced symptoms of muscle pain, fatigue and mild cough, while many infections are asymptomatic. Patients with severe infections may receive escalated treatment (e.g., mechanical ventilation or invasive mechanical ventilation), and reported higher levels of depression and anxiety (11), suggesting that severe symptoms may be associated with higher levels of depression and anxiety, and *vice versa*.

Therefore, although the new variant strain is more infectious, the symptoms caused by the infection are mild, and with the widespread vaccination and advanced treatment, the mortality is reduced. These evidences suggest that with the variation of SARS-CoV-2 strains, the psychological impact of the epidemic is gradually decreasing. However, the reports on the mental health status of patients infected with Omicron in Shanghai, China are rare. Therefore, we aim to explore the relation among depression, anxiety symptoms and perceived stress levels in COVID-19 patients who received treatment in Shanghai Fangcang shelter hospital.

Network analysis can be adopted to detect the symptom-to-symptom relationships, and the network model is useful to assess the importance of each symptom in a disorder (12–14). Currently, it is widely used to study the association between depression and anxiety in different populations during COVID-19 epidemic (13–17). As far as we know, few studies have adopted network analysis to explore the relationship between depression and anxiety symptoms in COVID-19 patients, especially when the number of asymptomatic patients is increased. The current study is focused on the COVID-19 patients with mild physical symptoms after Omicron infection. We hypothesized that compared with patients infected with previous strains (Delta strains etc.), the Omicron-infected patients, especially asymptomatic ones, have lower levels of anxiety, depression and perceived stress, and that possible changes exist in the structure of their networks. To test this hypothesis, we used network analysis to explore the relation between depression and anxiety symptoms in Omicron-infected patients. The following questions were asked: (1) Is the mental health level of the Omicron-infected patients lower that of the patients infected with other strains? (2) What are the most important symptoms in the network of depression, anxiety and perceived stress? (3) Which symptoms are the bridges between depression, anxiety and perceived stress networks? (4) Are there any network differences in gender, age, duration of symptoms and between the presence or absence of symptoms?

## Methods

### Ethics statement

This study has been reviewed and approved by the Medical Ethics Committee of the Department of Psychiatry, The 980th Hospital of PLA Joint Logistics Support Force (Project No.20200239). Participants provided verbal informed consent before participation in this study, and were informed that the survey was anonymous and the personal information would not be disclosed.

### Participants

Between April 15 and April 25, 2022, a total of 1305 patients with positive nucleic acid test results in Fangcang shelter hospital in Shanghai were included in this cross-sectional online survey. The inclusion criteria include: volunteer participation; access to devices such as mobile phone or tablet computer which can be used to answer the questionnaire; positive nucleic acid test results; and absence of symptoms or presence of mild symptoms. The exclusion criteria are incapability of answering the questionnaire; mental disorders; and symptom deterioration that requires isolation and additional care. The patients were invited by an experienced psychiatrist in Fangcang Hospital to scan the QR code through WeChat to obtain the questionnaire. The online questionnaire was presented and data were collected *via* the “Wenjuanxing” platform (www.wjx.cn). Before the survey, participants read the introduction to the questionnaire on the home page and submitted informed consent. For those who had difficulty in understanding the questionnaire, the investigator would provide necessary explanations. Those who filled in the questionnaire were considered willing to participate in the survey. Except for certain items such as age, other items were set up as mandatory, which means that only the participants who filled in those items are allowed to submit questionnaires. Participants aged below 18 or over 80 years (57 in total) and those who completed the questionnaire in <200 secs (nine in total) were excluded from the analysis.

### Measures

#### Depressive symptoms

This study adopted the Chinese version of Patient Health Questionnaire (PHQ-9) (18), which is widely used to assess depressive symptoms and its severity. There are nine items, including “Poor appetite, weight loss, or overeating” and “Feeling tired, or having little energy,” etc. Each participant was asked to choose the frequency of the event in the past 2 weeks. For each item, the score of ranges between 0 (“Not at all”) and 3 (“Nearly every day”). The total score is positively correlated with the severity of depression. In the current study, Cronbach' α for PHQ-9 was 0.89. Cut-off ≥ 10 was used to determine if the participants had depressive symptoms.

#### Anxiety symptoms

Anxiety symptoms were assessed using the Chinese version of seven-item Generalized Anxiety Disorder Scale (GAD-7) (19), which is widely used for assessing anxiety symptoms of participants within the last 2 weeks with high validity. For each item, the score ranges between 0 (“not at all”) and 3 (“nearly every day”), with the total score being 0 to 21. The total score is positively correlated with the depression severity. In the current study, Cronbach'α for GAD-7 was 0.92. Cut-off ≥ 10 was used to determine if the participants had anxiety symptoms.

#### Perceived stress

The short form Perceived Stress Scale (PSS-4) was adopted to assess patients' perceived stress (23), which is a widely used scale. The Chinese simplified version questionnaire contains four items (20), each of which is designed using a 5-point Likert scale (0 = never to 4 = very frequent), and the total score ranged from 0 to 16. The score is positively correlated with the severity of perceived stress. This scale has been proved to be reliable (21, 22). In the current study, Cronbach' α for PSS-4 was 0.73. Cut-off ≥ 8 was used to determine whether the participants perceive stress (23).

### Data analysis

Excel software was used to sort out data, and R software (version 4.0.3) and its software package was used for data analysis. Before network analysis, we used non-paranormal transformation to normalize all skewed data as previously described (24, 25).

We estimated the network structure of depression, anxiety and perceived stress (DAS) of the patients to identify which depression and anxiety symptoms were associated with perceived stress. As our data are ordinal variables, we used the extended Bayesian information criterion (EBIC) glasso function of the qgraph software package in R for estimating the network (26). As the weak connections may make the network complex and redundant, we used a graphical Least Absolute Shrinkage and Selection Operator (GLASSO) to shrink the weak edges and make small edges become zero-weight edges to obtain a sparse network structure. The estimated networks were undirected. The variables in the network are “nodes,” and the connections between the nodes are “edges,” which is usually known as partial correlation. In addition, the R package mgm was used to calculate the node predictability (27), which is considered to be the node variance that can be explained by all the neighboring nodes.

We used the qgraph package of R software for calculating the centrality index and visualize the network structure (28), and adopted the bootnet package to test the difference in node centrality (29). The expected influence centrality is the sum of edge weights (for example correlation coefficients) connected by each node, suggesting that if a certain symptom is activated, it may induce the activation of other symptoms (30). Negative correlation is represented by the red edge in the network structure, while positive correlation is represented by the blue edge. The thickness of an edge is positively correlated with the degree of relation and *vice versa*.

We used the bootnet function of R software for calculating the stability and accuracy of the network (29). First, we adopted the bootstrap method for evaluating the accuracy of edge weights by computing the 95% confidence interval (CI). If the CI was narrow, the edge weights and central indexes would be more accurately estimated. Then, we used the case-dropping bootstrap approach to assess the stability of the centrality index by calculation of the correlation stability coefficient (nboot = 2,000), which can be used to exclude the cases of the largest proportion when the correlation between the original centrality index and the subsample centrality index keeps to be at least 0.70 (95% probability). A previous study suggests that the correlation stability coefficient (CS) should remain higher than 0.25, better to be over 0.50 (29).

Bridge symptoms are considered to be the overlapping symptoms of two mental diseases (31). In this study, the R-package networktools was used to calculate the nodes where depressive symptoms, anxiety symptoms and perceived stress overlap with each other (32). Because the best index for identifying bridge nodes is the bridge expected influence centrality, we calculate the centrality of bridge expected influence. Bridge symptoms suggest that the risk of contagion to other communities is greater, and bridge symptom elimination could prevent the spreading between diseases (32). Here, we divided all nodes into three communities, with one containing nine depressive symptoms, one containing seven anxiety symptoms, and one containing four perceived stress nodes.

Finally, we used the Network Comparison Test function to know if there were differences in network structure and overall network connectivity (33). We included gender, a major variable for predicting depression and anxiety in COVID-19 patients (34), age (adult group: 18–59 years old and elderly group: 60–78 years old), presence of symptoms and duration of isolation in the network comparison. We calculated the global strength value and *p-*value of each network, as well as the number of edges with significant differences. Network structure refers to the biggest difference of corresponding edges between two networks, network edge invariance means the difference in individual edge weight between two networks, and global strength represents the weighted absolute sum of all edges on each network (33). Multiple comparisons between individual network edges were performed using Bonferroni-Holm correction (15).

## Results

### Descriptive statistics

A total of 1,239 patients infected with Omicron variant were included in the current research analysis, including 489 women, accounting for 39.47% of the total. The age of patients ranged from 18 to 78 (42.96 ± 13.10). A total of 480 infected patients (56.80% of the patients who reported symptoms) had symptoms such as cough, sore throat, fatigue, fever, and skin allergy, among which cough was one of the most common symptoms (Table 1). Based on the current criteria (score ≥ 10 for depression and anxiety or ≥ 8 for perceived stress), 9.03% had depression, 4.60% had anxiety, and 17.03% felt stressed. The average score of items was 0.07 to 0.59 (Table 2). Sleep problems were the most frequent symptoms, and suicidal ideation was the least frequent.

**Table 1:** Demographics of depression and anxiety severity of the participants (*n* = 1,239).

	**M (S.D.)/*n* (%)**
**Age**	42.96 *(13.10)*
Adult group (*n* = 1,127)	40.70 *(11.41)*
Elderly group (*n* = 112)	65.71 *(4.36)*
**Gender**	
Male	750 (60.53)
Female	489 (39.47)
**Symptoms**	
**Types of symptoms**	480 (38.74)
Cough	397 (82.71)
Pharyngalgia	208 (43.33)
Hypodynamic	113 (23.54)
Fever	33 (6.88)
Skin allergy	33 (6.88)
No symptoms	365 (29.46)
Missed	394 (31.80)
**Depression severity (total raw score)**	
0–4	913 (73.69)
5–9	214 (17.27)
10–14	76(6.13)
15–19	23 (1.86)
20–27	13 (1.05)
**Anxiety severity (total raw score)**	
0–4	1,013 (81.76)
5–9	169 (13.64)
10–14	38 (3.07)
15–21	19 (1.53)
**Perceived stress**	
≥8	211 (17.03)

The SD is in italics to distinguish it from %s in the other brackets.

**Table 2:** Abbreviation, mean, standard deviation, and presence of symptoms in PHQ-9, GAD-7, and PSS-4.

**Depression symptoms**	**Abbreviation**	**Mean (SD)**	**% Presence**	**Predictability**
Little interest or pleasure in doing things	D1: Anhedonia	0.47 (0.80)	32.93	0.58
Feeling down, depressed, irritable, or hopeless	D2: Sadness	0.34 (0.66)	26.31	0.55
Trouble falling asleep, staying asleep, or sleeping too much	D3: Sleeping	0.59 (0.90)	37.45	0.42
Feeling tired, or having little energy	D4: Fatigue	0.48 (0.75)	35.43	0.58
Poor appetite, weight loss, or overeating	D5: Appetite	0.38 (0.70)	28.57	0.42
Feeling bad about yourself, or that you're a failure or that you've let yourself or your family down	D6: Failure	0.28 (0.64)	19.85	0.53
Trouble concentrating on things like school work, reading, or watching TV	D7: Concentration	0.30 (0.65)	20.09	0.51
Moving or speaking so slowly that other people could notice	D8: Motor	0.20 (0.54)	15.17	0.53
Thinking that you would be better off dead, or of hurting yourself in some way	D9: Suicidality ideation	0.07 (0.35)	5.57	0.33
Feeling nervous, anxious, or on edge	A1: Nervousness	0.41 (0.70)	32.36	0.62
Not being able to stop or control worrying	A2: Uncontrollable worry	0.29 (0.62)	22.92	0.66
Worrying too much about different things	A3: Excessive worry	0.35 (0.67)	26.63	0.59
Trouble relaxing	A4: Trouble relaxing	0.29 (0.63)	22.20	0.66
Being so restless that it's hard to sit still	A5: Restlessness	0.23 (0.59)	16.95	0.69
Becoming easily annoyed or irritable	A6: Irritability	0.27 (0.59)	21.15	0.57
Feeling afraid as if something awful might happen	A7: Feeling afraid	0.22 (0.55)	16.79	0.56
Unable to control the important things in your life	S1: Uncontrollable	0.60 (0.98)	31.64	0.54
Not confident about your ability to handle your personal problems	S2: Not confident	1.26 (1.43)	49.15	0.50
Things were not going your way	S3: Unsatisfactory	1.05 (1.27)	47.05	0.47
Difficulties were pilling up so high that you could not overcome them	S4: Many difficulties	0.69 (1.02)	36.56	0.52

D1–D9 are PHQ-A items on depression. A1–A7 are GAD-7 items on anxiety. S1–S4 are PSS-4 items on perceived stress. SD, Standard Deviation.

### Network structure

**Figure 1:**
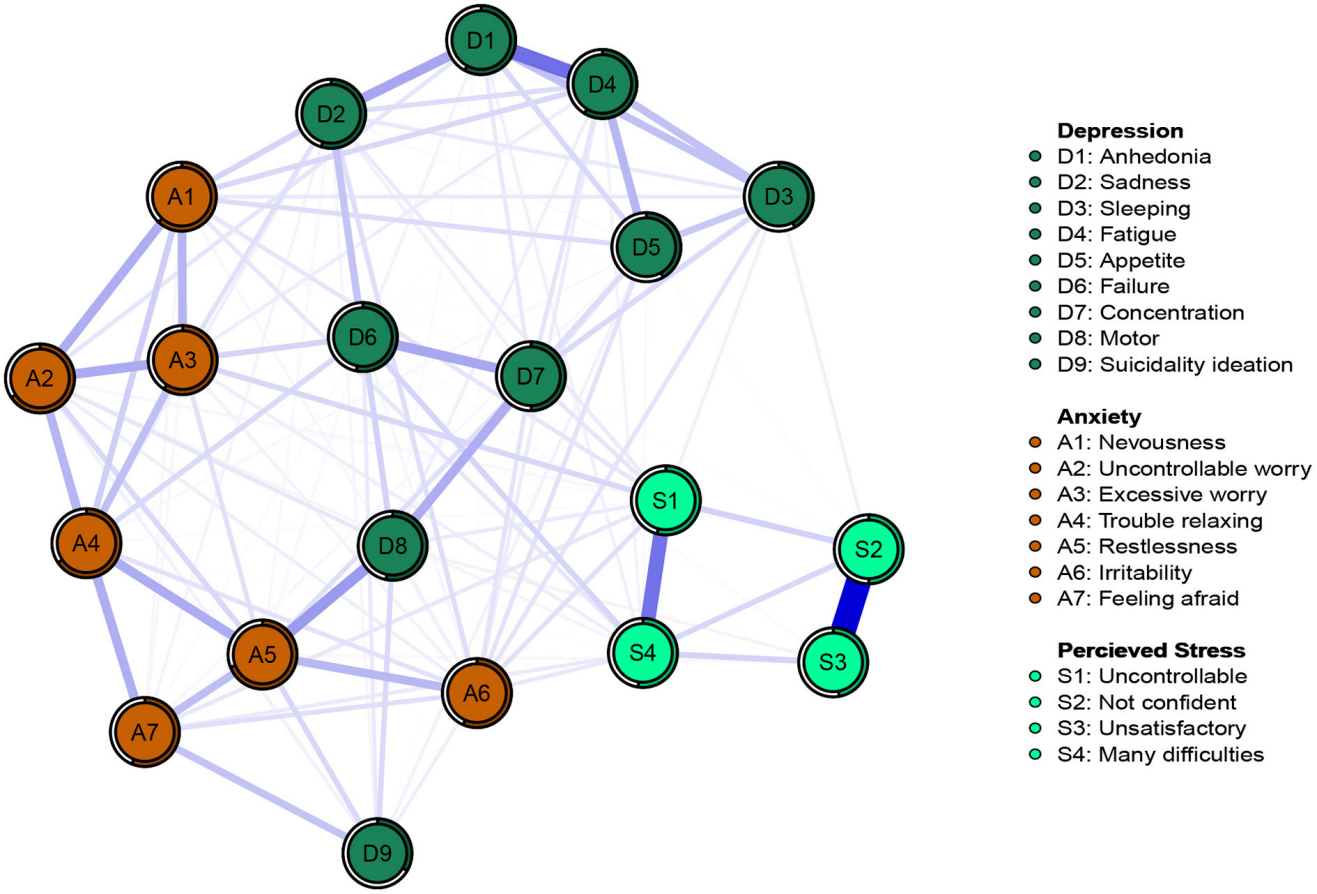
Network structure of depression, anxiety, and perceived stress in patients infected with Omicron.

The network structures of DAS were shown in Figure 1. Among the 190 edges with possible connections, 121 were not zero (63.68%). The edges with the strongest connections in the network were S2 (not confident)-S3 (unsatisfactory) (weight = 0.62), D1 (anhedonia)-D4 (fatigue) (weight = 0.35), S1 (uncontrollable)-S4 (many difficulties) (weight = 0.34), and D8 (motor)-A5 (restlessness) (weight = 0.24) in a descending order. Figure 1 shows predictability through a ring around the nodes. The nodes had a predictability ranging from 0.33 to 0.69, and the average predictability was 0.54 (Table 2). The bootstrapped 95% CI was relatively narrow; thus, the edges of the DAS network are accurate (Supplementary Figure 1). Supplementary Figure 2 showed the bootstrapped difference test for edge weights.

### Centrality of network

**Figure 2:**
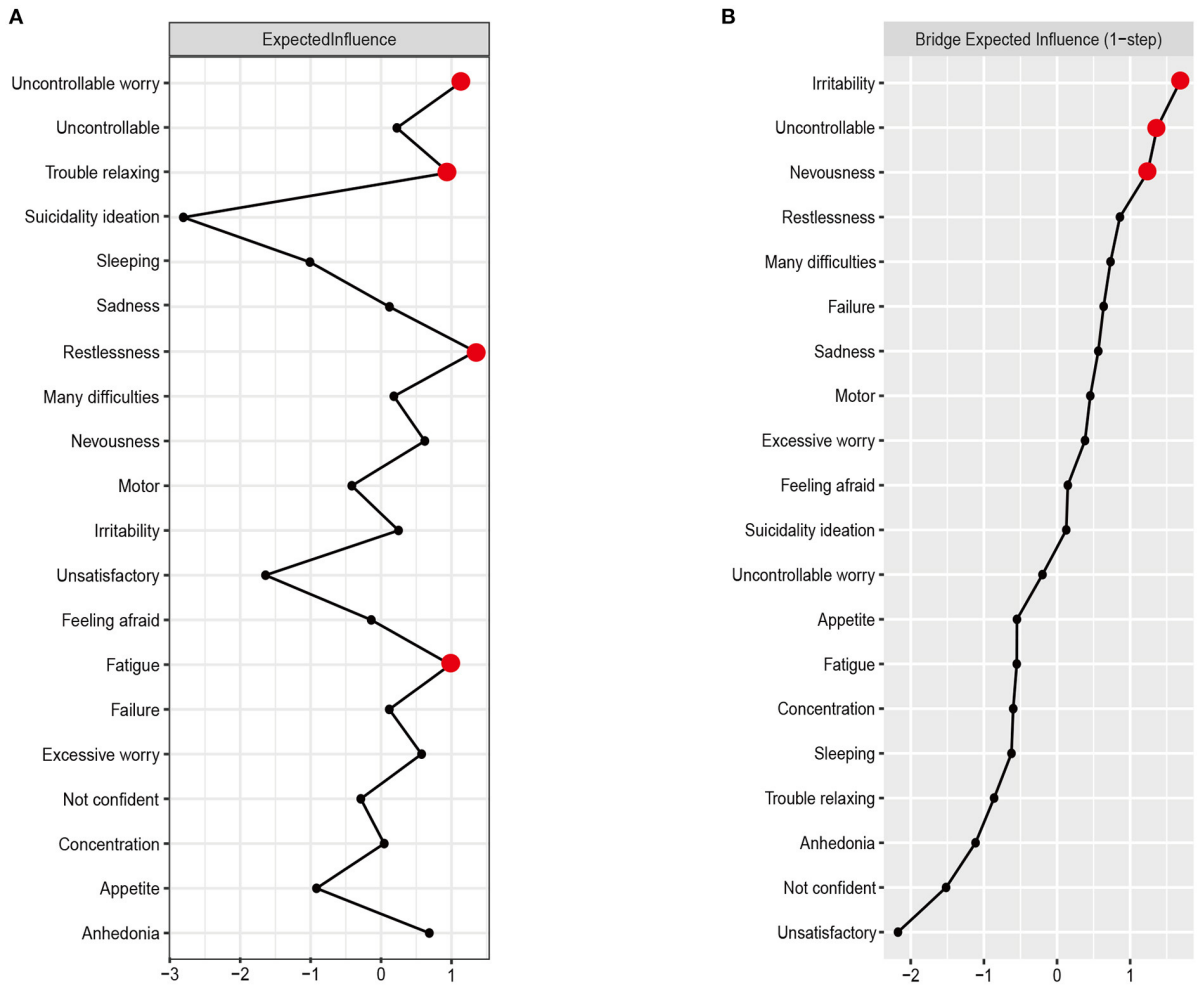
Centrality plot of the expected influence **(A)** and bridge expected influence **(B)** of depression, anxiety and perceived stress nodes in the network (z-score).

The expected influence centrality of DAS network is presented in Figure 2A. In DAS network, the node expected influence of anxiety symptom A5 (restlessness) was the highest, followed by A2 (uncontrollable worry), A4 (trouble relaxing) and depression symptom D4 (fatigue). Perceived stress node S3 (unsatisfactory) had the lowest node expected influence in the DAS network. The CS-coefficient of stability of the expected influence centrality index was 0.75 (Supplementary Figure 3), higher than 0.5, the recommended critical value (29). Supplementary Figure 4 showed the bootstrapped difference test for node expected influence.

### Bridge nodes

The bridge expected influence centrality of DAS network is presented in Figure 2B. Anxiety symptom A6 (irritability) had the highest node expected influence in DAS network, followed by perceived stress node S1 (uncontrollable) and anxiety symptom A1 (nervousness). The correlation stability coefficient of the bridge expected-influence was 0.75 (Supplementary Figure 5), suggesting that the bridge expected influence has an acceptable stability. The bootstrapped difference test for node bridge expected influence is shown in Supplementary Figure 6.

### Network comparison analysis

The network structures of four sub-sample are shown in Supplementary Figure 7. Significant differences existed in the DAS networks between patients with or without physical symptoms, but the global strength between them was not significantly different (network variance [M] = 0.28, *P* = 0.03; Global strength variance [S] = 0.17, no physical symptoms = 9.23, physical symptoms = 9.06, *P* = 0.39). Thus, a differences exist in the interaction between items, but not in the overall connectivity of items in the network. There may be differences in central symptoms between patients with or without physical symptoms. The network structure of the two groups was further analyzed, and the results showed that the central symptoms of those with physical symptoms were A4 (trouble relaxing) (EI = 1.43), while those without physical symptoms were A2 (uncontrollable worry) (EI = 2.24). No difference was found in the indexes of gender subsample (network variance [M] = 0.17, *P* = 0.57; global strength variance [S] = 0.24, male = 9.59, female = 9.84, *P* = 0.29), age group subsample (network variance [M] = 0.24, *P* = 0.62; Global strength variance [S] = 0.11, adult group = 9.21, Elderly group = 9.10, *P* = 0.74), and isolation duration subsample (network variance [M] = 0.23, *P* = 0.45; Global strength variance [S] = 0.05, about 1 week = 9.18, more than 2 weeks = 9.13, *P* = 0.83).

## Discussion

To our knowledge, this is the first study to explore the complex networks of depression, anxiety symptoms and perceived stress in Omicron-infected patients. We first analyzed the depression, anxiety and perceived stress levels of these patients, then analyzed the bridge expected influence through network analysis, and finally compared the network differences of subsamples of gender, age, isolation time and the presence of somatic symptoms.

The results of descriptive analysis showed that the levels of depression, anxiety and perceived stress in the patients in Fangcang shelter hospital in Shanghai, China were significantly lower than those in the inpatients in Wuhan from February 15 to February 29, 2020 (35, 36) (depression: 24.71–54.45 vs. 9.03%; anxiety: 16.47–50.11 vs. 4.60%, perceived stress: 39.70 vs. 17.03%). Possible explanations are listed as follows. First, the infection level of the patients in the two samples is different. The patients with COVID-19 in Wuhan, China from January to March 2020 mainly had moderate or severe infection, while only half (56.80%) of the patients in the current study had mild symptoms (cough, etc.). Second, with the improvement of vaccination, COVID-19 screening, and treatment methods, and the increased public awareness of COVID-19, the virulence of the virus is weakened and the fear about COVID-19 infection is reduced (37). This is consistent with our hypothesis that the mental health level of the Omicron-infected patients is higher than that of the patients infected with prior strains, and that mild symptoms of COVID-19 infection are associated with low level of depression, anxiety, and perceived stress.

In the current network structure, the edge with the strongest connection is within the same disorder, consistent with the results of previous network analyses (15, 38–40). The edge with the strongest connection in the network is S2 (not confident)—S3 (unsatisfactory), which belongs to the “perceived self-efficacy” dimension in PSS. In addition, a strong connection also existed between nodes S1 (uncontrollable)-S4 (many difficulties) of another dimension “perceived helplessness” in the PSS, similar to the previous perceived stress network (41). S2 (not confident)—S3 (unsatisfactory) was the edge with the strongest connection in the perceived stress items in the trauma and perceived stress networks instead of S1 (uncontrollable)-S4 (many difficulties). The possible explanation is that during the isolation due to COVID-19 infection, as individuals are overwhelmed by a bunch of events, such as the time of recovery and inability to care for their families, they developed a sense of low self-efficacy and helplessness (42), which may affect their mental health (43).

We also found a strong connection between depression symptoms such as D1 (anhedonia)–D4 (fatigue). The previous network analysis of female nurse students, who were not infected with SARS-CoV-2, showed a strong connection between depression symptoms D3 (sleeping difficulty) and D4 (fatigue) and D1 (anhedonia) and D4 (fatigue) (17). Among the college students in the late stage of the epidemic, the edges with the strongest connection were A1 (nervousness worry)-A2 (uncontrollable) and A2 (uncontrollable worry)-A3 (excessive worry) (15). Among Filipino migrant workers, D7 (concentration difficulties)-D8 (psychomotor aging/retirement), and D3 (sleep difficulties)—D4 (fatigue) and A3 (excessive worry)—A4 (trouble relaxing) and D8 (psychomotor aging/retirement)—D9 (thoughts of death) were the edges with the strongest connection (44). These evidences show that differences and similarities exist in ways of interactions between Omicron-infected and non-infected patients. The most possible explanation may be that although the patients with Omicron infection have mild pathological symptoms, they have to receive treatment in isolation. On the one hand, the treatments they received increase their fatigue; on the other hand, the limited range of activities results in loss of pleasure. Therefore, measures such as providing timely and reasonable treatment information to patients, improving their sense of control over treatment, reducing fatigue, and appropriately increasing recreational activities may help alleviate anxiety, depression, and perceived stress severity.

The results of the node expected influence centrality of nodes showed that the expected influence value of anxiety symptom A5 (restlessness) was the highest in the network, indicating that it plays an important role in depression, anxiety and perceived stress network. In addition, A2 (uncontrollable worry) and A4 (trouble relaxing) were also the main central symptoms, consistent with the results of Chinese doctors in the post epidemic period (45). On the one hand, due to the different physique of individuals, their symptoms and recovery speed after the infection may differ from one another. On the other hand, because of the pandemic of Omicron, we need to continue to implement strict public health measures. These uncertainties may increase the anxiety level of the patients (42). D4 (fatigue) also had a strong expected influence value, consistent with the results of previous studies conducted in college students (15, 17), which may be explained by more physical examinations, worse sleep quality and less exercises during COVID-19 isolation. These evidences suggest that these symptoms play a major role in activating and maintaining depression, anxiety and perceived stress networks in patients infected with Omicron.

The centrality of the bridge expected influence of the node showed that A6 (irritability) was a bridge symptom in the network, consistent with the results of studies conducted in Chinese doctors and nurse students during the epidemic (17, 45), but inconsistent with the results of study in general college students. In the later stage of the epidemic, the bridge symptom of college students was D8 (motor) (15). We propose two possible explanations. First, medical staff have time-space contact with patients, which increases the risk of exposure to novel coronavirus. Like the patients, they cannot control the development of the epidemic, and have to be exposed to the risk of infection due to their work environment. These uncertainties make them prone to irritability. Second, the general population has a lower risk of infection with SARS-CoV-2 because isolation restricts their movement. In addition, we also found that perceived stress S1 (uncontrollable) was the second bridge symptom in the network. Previous studies have shown that the level of perceived stress in COVID-19 patients is as high as 39.7% (42), suggesting that patients perceived stress because they lose control of many things, resulting in depression or anxiety. It is worth noting that the current network is undirected; therefore, it is necessary to further study the sequence of developing these symptoms in order to intervene the initial symptoms with more accurate means. Therefore, intervention against bridge symptoms can effectively prevent the connection between symptoms.

The comparison of the network for the four sub-samples showed no difference between them except for the network with physiological symptoms. The core symptom in the DAS network of patients with physiological symptoms was A4 (trouble relaxing), and the core symptom in the DAS network of asymptomatic patients was A2 (uncontrollable worry). The presence of physiological symptoms can predict the disease risk of patients after infection. Previous studies have shown that patients with infection symptoms have higher levels of depression and anxiety than asymptomatic patients (42), and the level of depression increases with the severity of physiological symptoms (8), suggesting different targets of intervention for symptomatic and asymptomatic patients.

Notably, our current study found no differences in the DAS network between men and women, inconsistent with the results of some previous studies (46). A possible explanation is that gender differences in depression and anxiety symptoms only emerge when the prevalence of depression and anxiety is high. Generally, in children, the prevalence of depression and anxiety is lower and there are no gender differences whereas in the post-pubertal group, the prevalence of depression and anxiety increases and gender differences exist (47). A recent review of gender differences in depression (48) suggests that women typically have a higher prevalence of depression but less severe symptoms than men, which may explain why gender differences exist when the prevalence of depression is high and are reduced when the prevalence of depression is low. This evidence suggests that during depression and anxiety pandemic, treatment should be attentive to the gender differences.

This study has several limitations. First, as no surveys were conducted to investigate whether the patients have basic diseases, it is unclear whether comorbidity exists, which may affect the popularization of the research results. Second, the cross-sectional questionnaire survey is easily affected by the current status of the respondents, and cannot reflect the changes in the symptoms. In addition, this study lacks a strict experimental design to compare the differences in symptoms before and after COVID-19 infection. Therefore, it is difficult to prove that these symptoms are completely caused by COVID-19 infection. Finally, this study did not validate the proposed intervention targets, which requires follow-up research to further verify the effectiveness of these targets for intervention.

## Conclusion

In conclusion, Omicron-infected patients isolated in Fangcang shelter hospital in Shanghai have reduced levels of depression, anxiety symptoms and perceived stress. Network analysis found that central symptoms A5 (restlessness), A2 (uncontrollable worry), A4 (trouble relaxing) and D4 (fatigue) and bridge symptoms A6 (Irritability) and S1 (uncontrollable) are potential targets for intervention. With the continuous evolution of SARS-CoV-2, the number of patients with mild or asymptomatic infection is growing. Therefore, it is important to take targeted interventions (such as relaxation therapy and cognitive behavioral therapy) against depression, anxiety and perceived stress in these patients. Future study needs to consider the cause-effect relation between the physical symptoms and mental health of the COVID-19 patients, and validate the efficacy of intervention target.

## Data availability statement

The raw data supporting the conclusions of this article will be made available by the authors, without undue reservation. Requests to access these datasets should be directed to KL, risyaiee@msn.cn.

## Ethics statement

The studies involving human participants were reviewed and approved by Medical Ethics Committee of the Department of Psychiatry, The 980th Hospital of PLA Joint Logistics Support Force (Project No. 20200239). The patients/participants provided their written informed consent to participate in this study.

## Author contributions

KuL: data curation, writing—original draft, formal analysis, and writing—review & editing. KeL: software, data curation, and writing—review & editing. LR: software, data curation, writing—original draft, and writing—review & editing. XZ, CL, XL, LW, and ZF: writing—review & editing. LL: data curation, writing—original draft, and writing—review & editing. All authors contributed to the article and approved the submitted version.

## Funding

This study was supported by the Health Commission of Hebei Provincial (No.20200239) and the fund was used for software development in the research to provide survey results reports for survey participants and also played a role in data analysis.

## Conflict of interest

The authors declare that the research was conducted in the absence of any commercial or financial relationships that could be construed as a potential conflict of interest.

## Publisher's note

All claims expressed in this article are solely those of the authors and do not necessarily represent those of their affiliated organizations, or those of the publisher, the editors and the reviewers. Any product that may be evaluated in this article, or claim that may be made by its manufacturer, is not guaranteed or endorsed by the publisher.
