# Supplementary material for: Adjusting intervention strategies for mental health of COVID-19 patients: A network analysis based on a survey in Omicron-infected patients
Source: Front Public Health. 2022 Nov 17;10:1038296. doi: 10.3389/fpubh.2022.1038296 (PMC9714331; doi:10.3389/fpubh.2022.1038296)
Supplement: Supplementary file 1 [file Data_Sheet_1.docx]

**Supplementary Material**

Adjusting intervention strategies for mental health of COVID-19 patients: a network analysis based on a survey in Omicron-infected patients

1. Figure 1. Accuracy of edge weights of DAS network
2. Figure 2. Bootstrapped difference test for edge weights of DAS network
3. Figure 3. Stability of node expected influences of DAS network
4. Figure 4. Bootstrapped difference test for node expected influences of DAS network
5. Figure 5. Stability of node bridge expected influences of DAS network
6. Figure 6. Bootstrapped difference test for node bridge expected influences of DAS network
7. Figure 7. Network comparisons of DAS by genders, presence of symptoms and duration of isolation in patients with Omicron


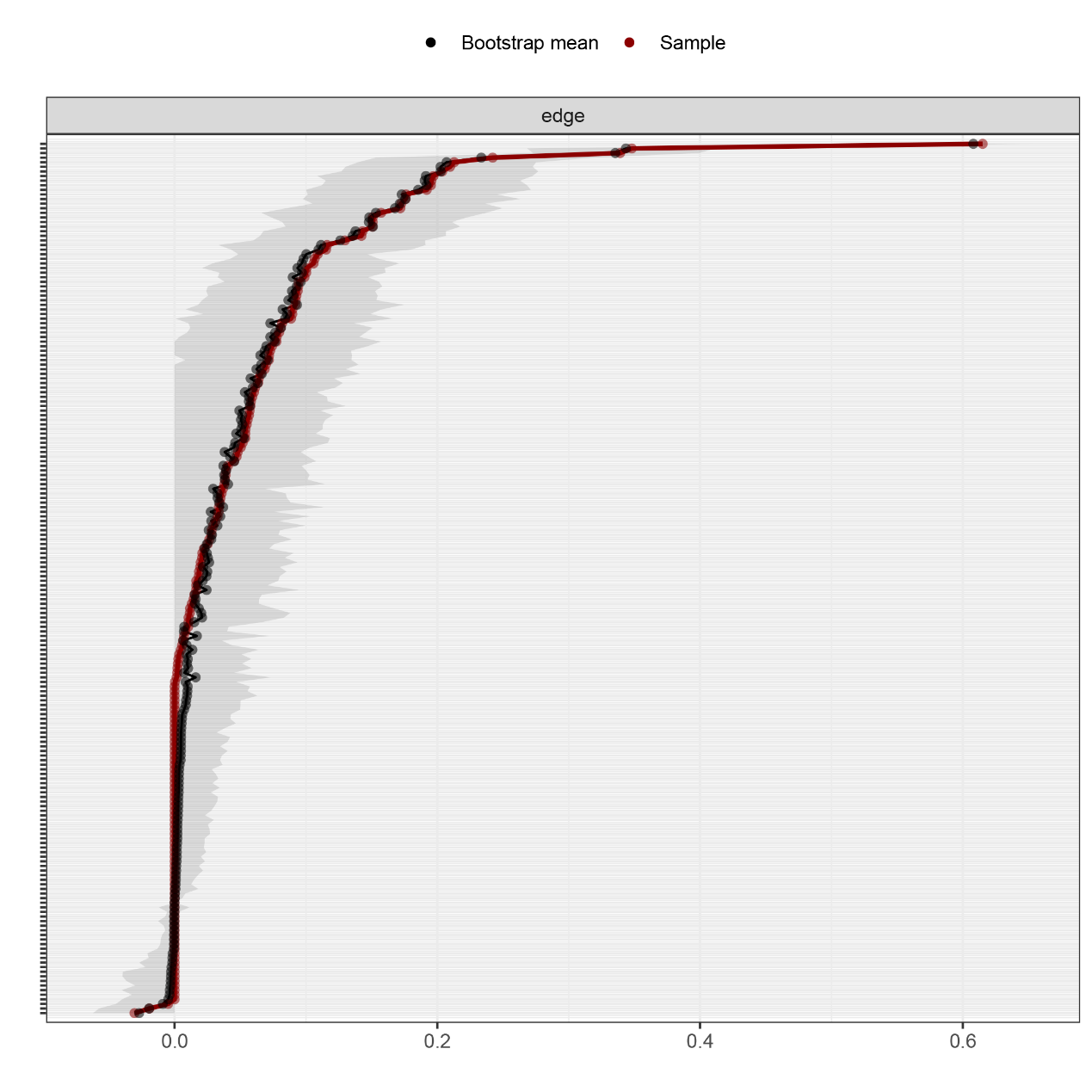


Figure 1. Accuracy of edge weights of DAS network

*Note*: The red line depicts the sample edge weights and the gray bar depicts the bootstrapped confidence interval.


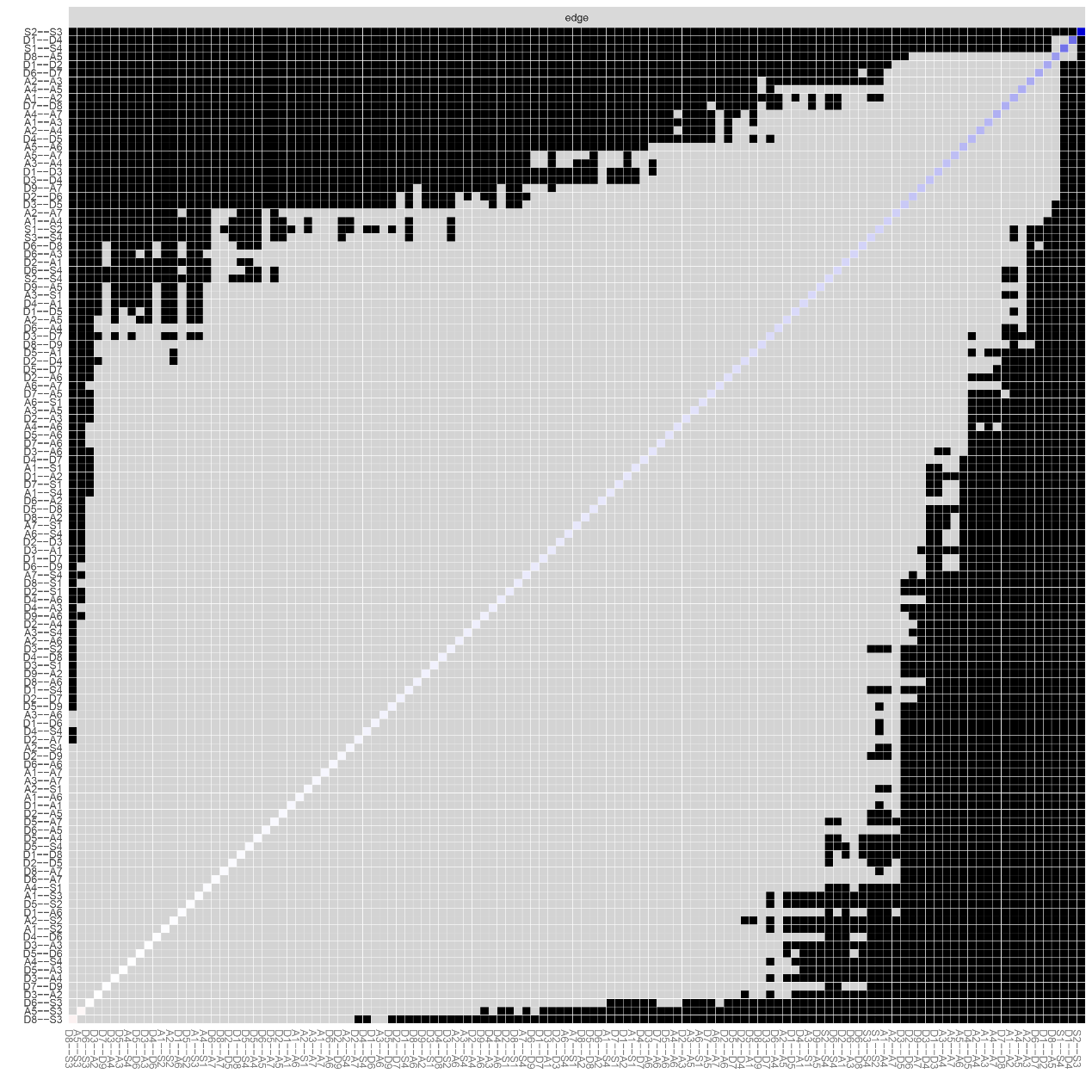


Figure 2. Bootstrapped difference test for edge weights of DAS network

*Note*: Gray boxes indicate edge weights that do not differ significantly from one another, while black boxes indicate edge weights that do differ significantly. Blue and red boxes on the diagonal correspond to edge weights with positive and negative correlations, respectively.


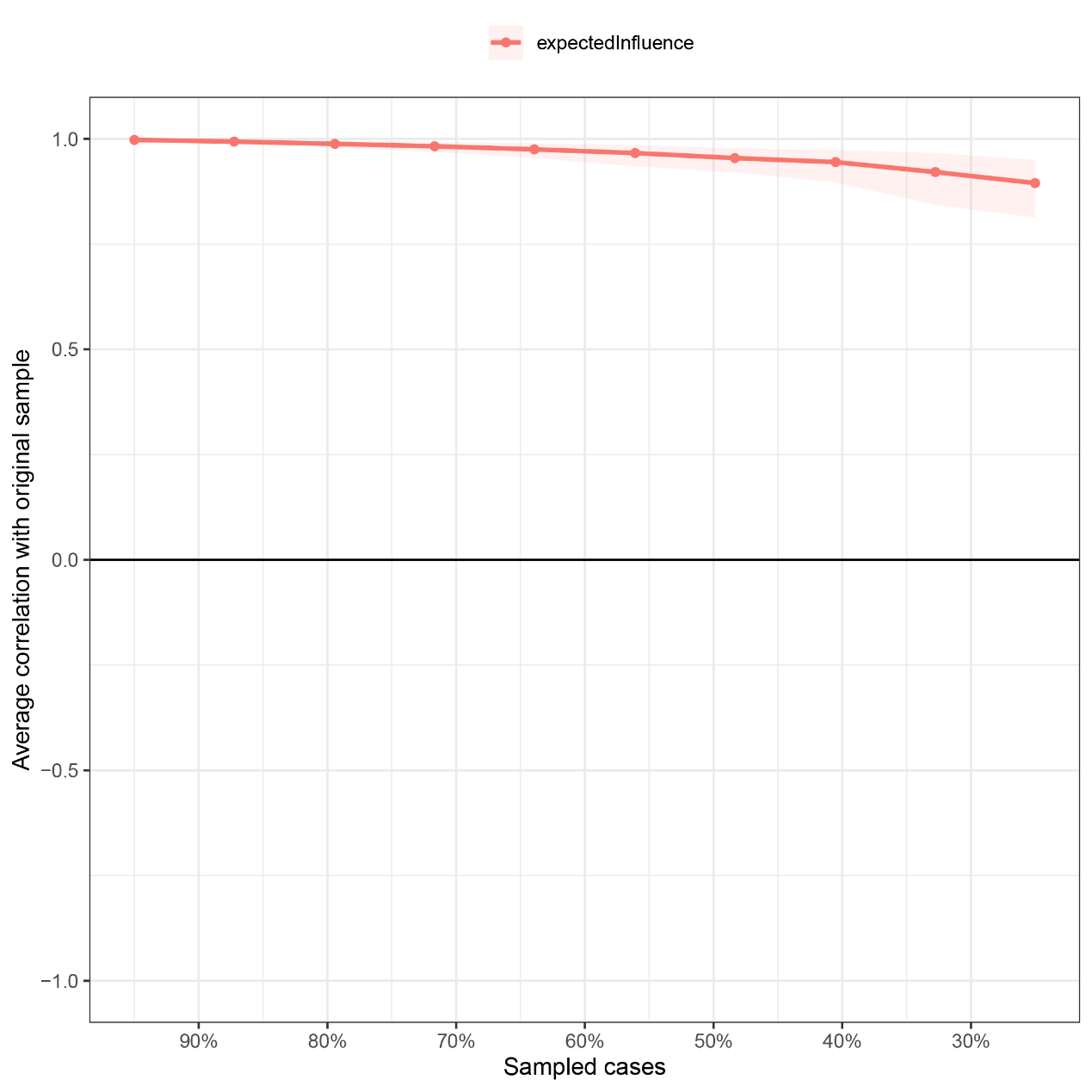


Figure 3. Stability of node expected influences of DAS network

*Note*: The red line represents the average correlation between node expected influences in the full sample and subsample with the red area depicting the 2.5th quantile to the 97.5th quantile.


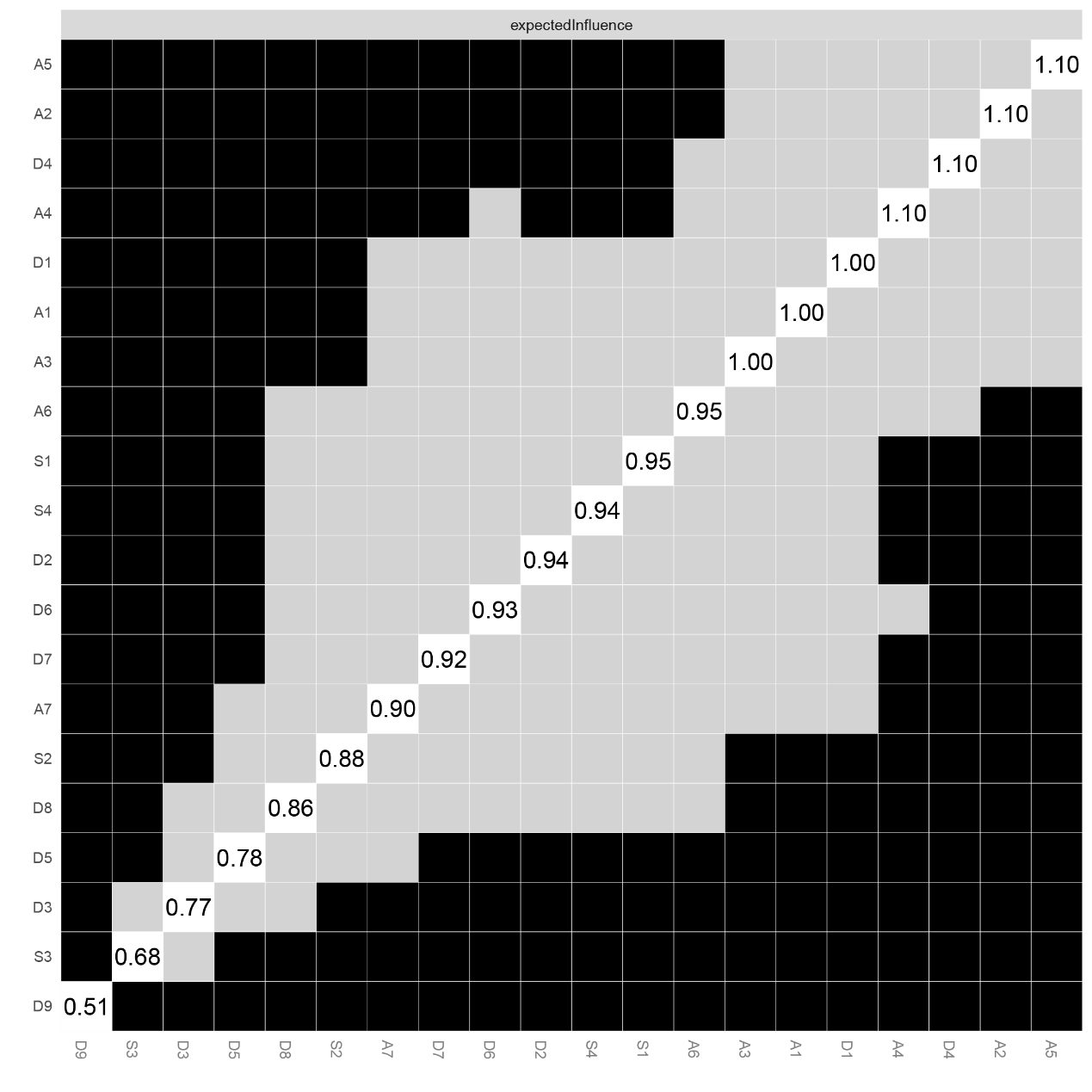


Figure 4. Bootstrapped difference test for node expected influences of DAS network

*Note*: Gray boxes indicate node expected influences that do not differ significantly from one another, while black boxes indicate node expected influences that do differ significantly.


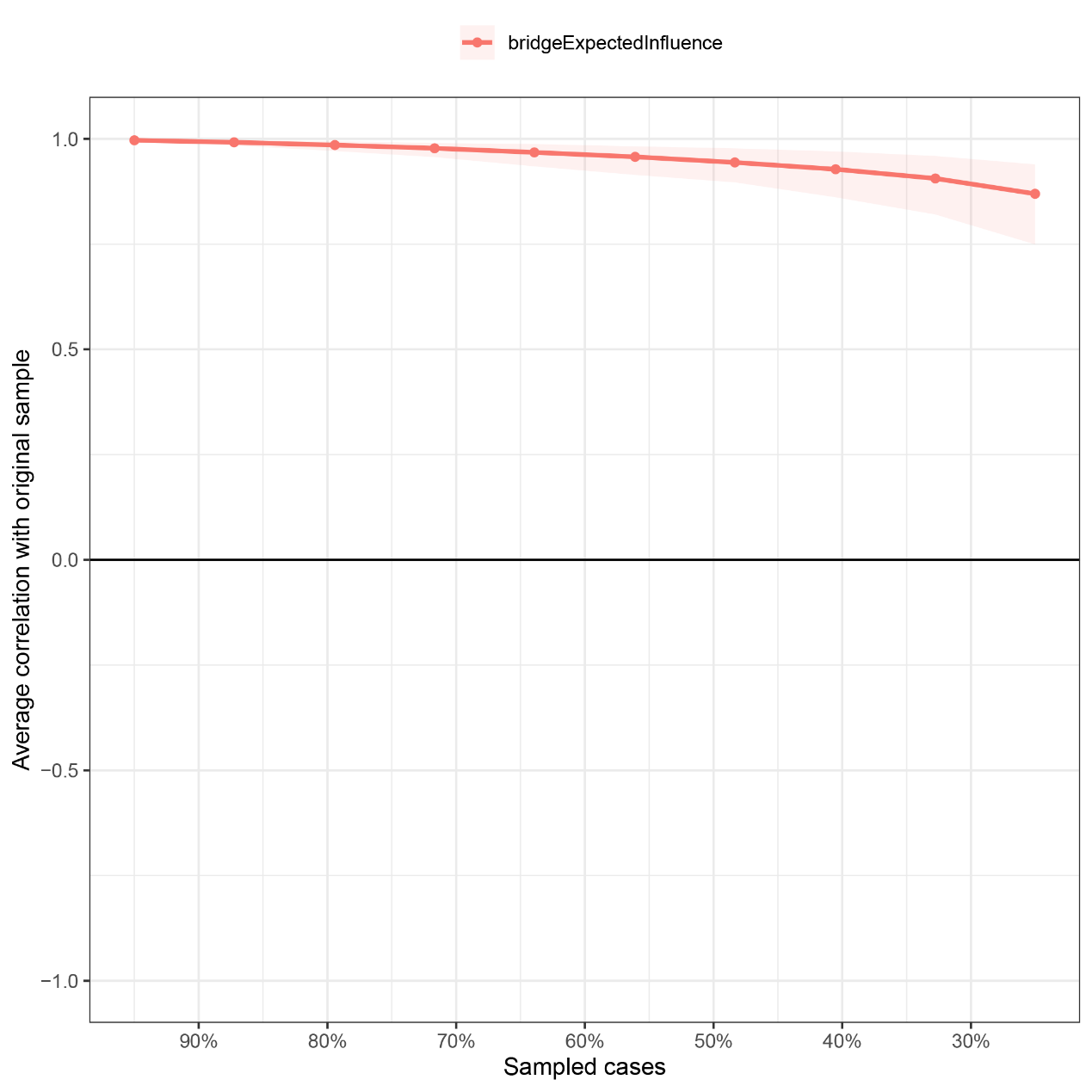


Figure 5. Stability of node bridge expected influences of DAS network

*Note*: The red line represents the average correlation between node bridge expected influences in the full sample and subsample with the red area depicting the 2.5th quantile to the 97.5th quantile.


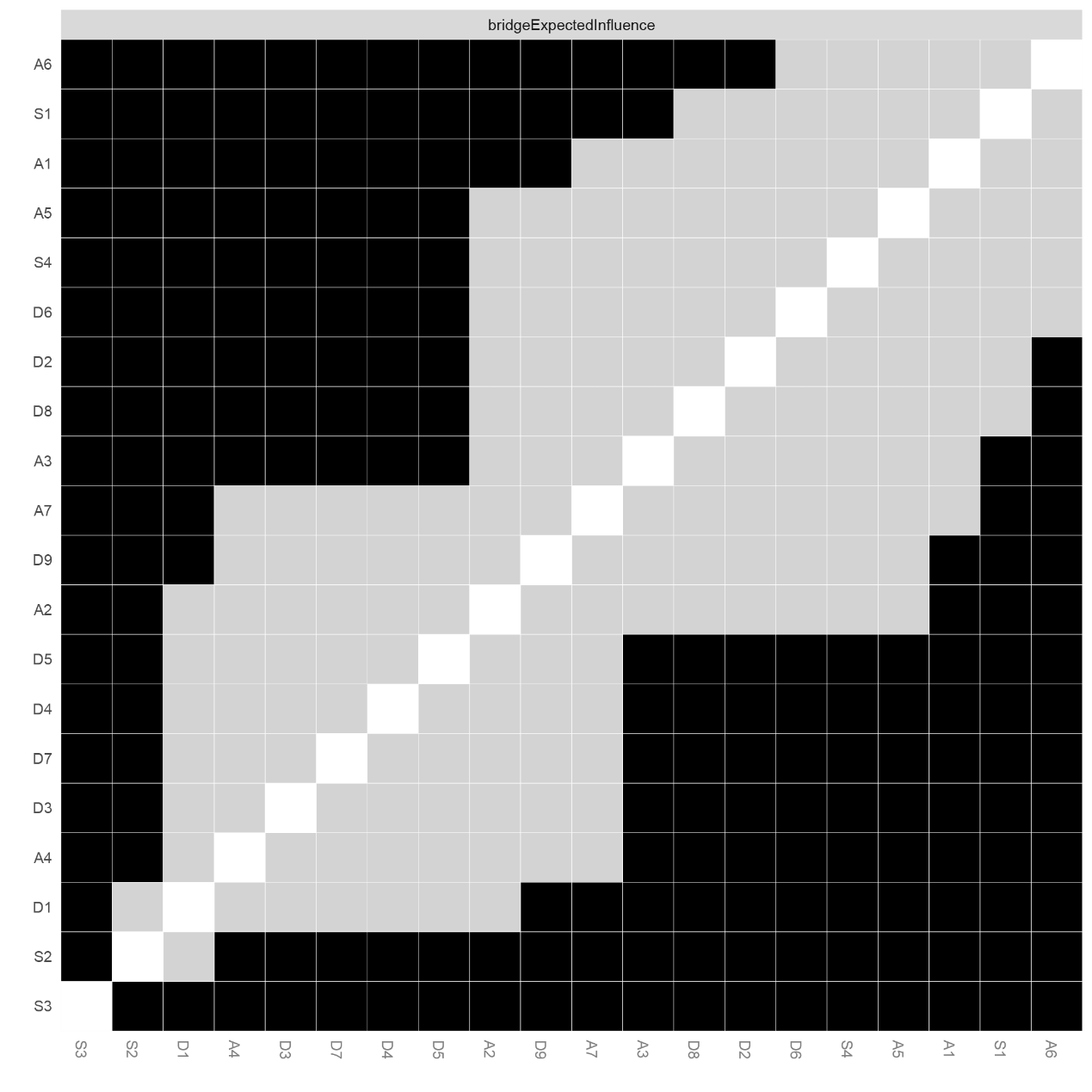


Figure 6. Bootstrapped difference test for node bridge expected influences of DAS network

*Note*: Gray boxes indicate node bridge expected influences that do not differ significantly from one another, while black boxes indicate node bridge expected influences that do differ significantly.


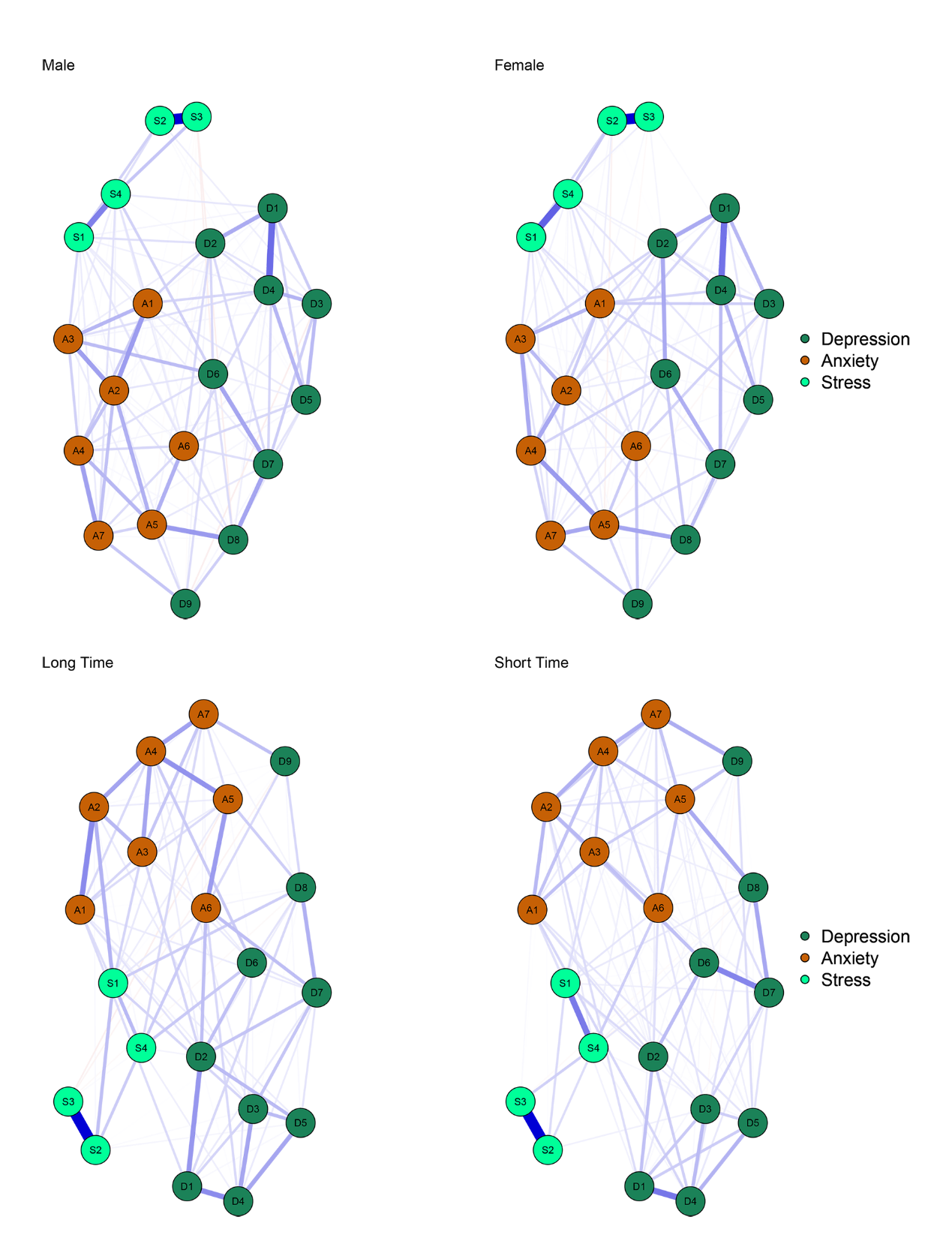


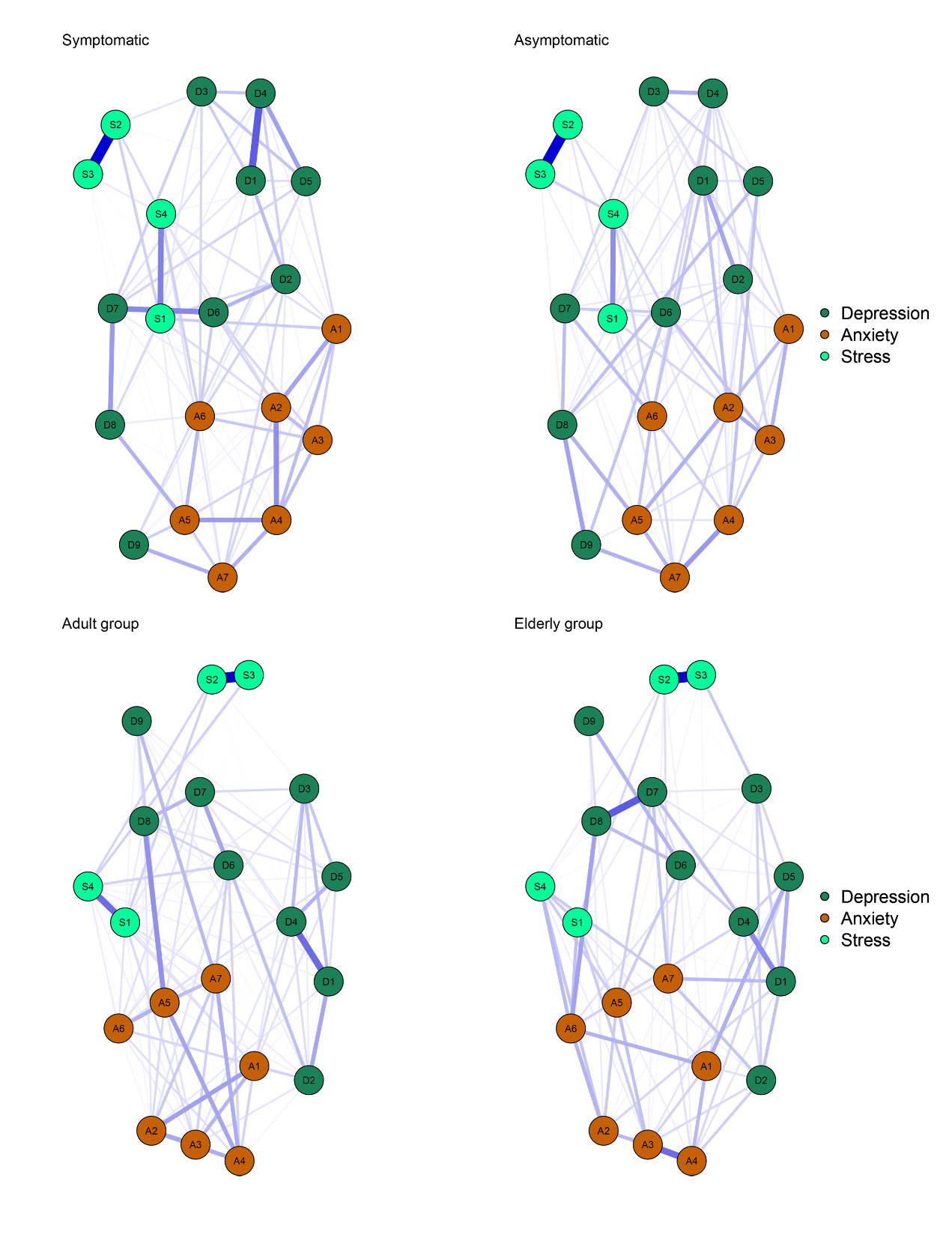


Figure 7. Network comparisons of DAS by genders, duration of isolation, presence of symptoms and age in patients with Omicron
